# Suppression of GHS-R in AgRP Neurons Mitigates Diet-Induced Obesity by Activating Thermogenesis

**DOI:** 10.3390/ijms18040832

**Published:** 2017-04-14

**Authors:** Chia-Shan Wu, Odelia Y. N. Bongmba, Jing Yue, Jong Han Lee, Ligen Lin, Kenji Saito, Geetali Pradhan, De-Pei Li, Hui-Lin Pan, Allison Xu, Shaodong Guo, Yong Xu, Yuxiang Sun

**Affiliations:** 1 Department of Nutrition and Food Science, Texas A&M University, College Station, TX 77843, USA; cjwu@tamu.edu (C.-S.W.); shaodong.guo@tamu.edu (S.G.); 2 United States Department of Agriculture/Agriculture Research Service Children’s Nutrition Research Center, Department of Pediatrics, Baylor College of Medicine, Houston, TX 77030, USA; ngalayuh@yahoo.com (O.Y.N.B.); jingyue7488@yahoo.com (J.Y.); jhleecw@gmail.com (J.H.L.); ligenlin1979@gmail.com (L.L.); kenji-saito@uiowa.edu (K.S.); gpradhan@bcm.edu (G.P.); yongx@bcm.edu (Y.X.); 3 Reproductive Medical Center, Tongji affiliated Hospital of Tongji Medical College, Huazhong University of Science and Technology, Wuhan 430030, China; 4 College of Pharmacy, Gachon University, Incheon 21936, Korea; 5 State Key Laboratory of Quality Research in Chinese Medicine, Institute of Chinese Medical Sciences, University of Macau, Macao 999078, China; 6 Interdepartmental Program in Translational Biology and Molecular Medicine, Baylor College of Medicine, Houston, TX 77030, USA; 7 Division of Anesthesiology and Critical Care, University of Texas MD Anderson Cancer Center, Houston, TX 77030, USA; dpli@mdanderson.org (D.-P.L.); huilinpan@mdanderson.org (H.-L.P.); 8 Diabetes Center, University of California, San Francisco, CA 94143, USA; Allison.Xu@ucsf.edu; 9 Huffington Center on Aging, Baylor College of Medicine, Houston, TX 77030, USA

**Keywords:** Agouti-related peptide (AgRP), ghrelin, growth hormone secretagogue receptor (GHS-R), diet-induced obesity (DIO), thermogenesis

## Abstract

Ghrelin, an orexigenic hormone released primarily from the gut, signals the hypothalamus to stimulate growth hormone release, enhance appetite and promote weight gain. The ghrelin receptor, aka Growth Hormone Secretagogue Receptor (GHS-R), is highly expressed in the brain, with highest expression in Agouti-Related Peptide (AgRP) neurons of the hypothalamus. We recently reported that neuron-specific deletion of GHS-R completely prevents diet-induced obesity (DIO) in mice by activating non-shivering thermogenesis. To further decipher the specific neuronal circuits mediating the metabolic effects of GHS-R, we generated AgRP neuron-specific GHS-R knockout mice (*AgRP-Cre*;*Ghsr^f/f^*). Our data showed that GHS-R in AgRP neurons is required for ghrelin’s stimulatory effects on growth hormone secretion, acute food intake and adiposity, but not for long-term total food intake. Importantly, deletion of GHS-R in AgRP neurons attenuated diet-induced obesity (DIO) and enhanced cold-resistance in mice fed high fat diet (HFD). The HFD-fed knockout mice showed increased energy expenditure, and exhibited enhanced thermogenic activation in both brown and subcutaneous fat; this implies that GHS-R suppression in AgRP neurons enhances sympathetic outflow. In summary, our results suggest that AgRP neurons are key site for GHS-R mediated thermogenesis, and demonstrate that GHS-R in AgRP neurons plays crucial roles in governing energy utilization and pathogenesis of DIO.

## 1. Introduction

Ghrelin signaling has profound effects on energy- and glucose-homeostasis [1]. Ghrelin is produced predominantly by enteroendocrine cells in the gastric oxyntic mucosa [2,3,4], and is the only orexigenic hormone known to stimulate growth hormone (GH) release, enhance feeding and promote weight gain [1,2,4,5,6,7]. Ghrelin’s biological effects are mediated through the growth hormone secretagogue receptor (GHS-R) [1,3], and activation of GHS-R increases food intake and body weight [2,3,4]. It has been reported that global deletion of GHS-R protects against diet-induced obesity (DIO) [8], and we have shown that global deletion of GHS-R alleviates age-associated obesity and insulin resistance [9,10,11]. Moreover, we recently reported that GHS-R deletion in all neurons completely prevents DIO and attenuates DIO-associated insulin resistance, exhibiting activated thermogenesis and enhanced physical activity [12]. While the new finding is very exciting, the specific neurons associated with GHS-R mediated metabolic effects have remained unclear.

GHS-R is primarily expressed in the brain, and at lower expression levels in peripheral tissues such as pancreas, muscle and adipose tissues [5,13,14,15]. In the brain, the highest expression of GHS-R is detected in the arcuate nucleus (ARC) in hypothalamus, particularly in orexigenic neuropeptide Y (NPY)/Agouti-Related Peptide (AgRP) neurons [16,17]. The NPY/AgRP neurons reside along with the anorexigenic neurons expressing proopiomelanocortin (POMC) in the ARC. The activity of AgRP and POMC neurons are regulated by hormones such as ghrelin, leptin and insulin and by nutrients such as glucose [18,19,20]. During food deprivation, AgRP neurons are activated by ghrelin to promote hunger and feeding [5,21,22,23,24,25,26,27,28,29]. Recently, AgRP neurons have been reported to regulate adaptive thermogenesis and browning of white adipose tissue (WAT) [18]. However, it is unknown whether AgRP neurons are important in mediating GHS-R suppression-associated thermogenic activation.

In this study, we investigated the specific role of GHS-R in AgRP neurons in regulating thermogenesis. We used a novel mouse model in which GHS-R was selectively deleted from AgRP neurons (*AgRP-Cre*;*Ghsr^f/f^*); we then studied the mice under regular chow- and HFD-feeding and under cold challenge.

## 2. Results

### 2.1. Generation of AgRP-Cre;Ghsr^f/f^ Mice

**Figure 1:**
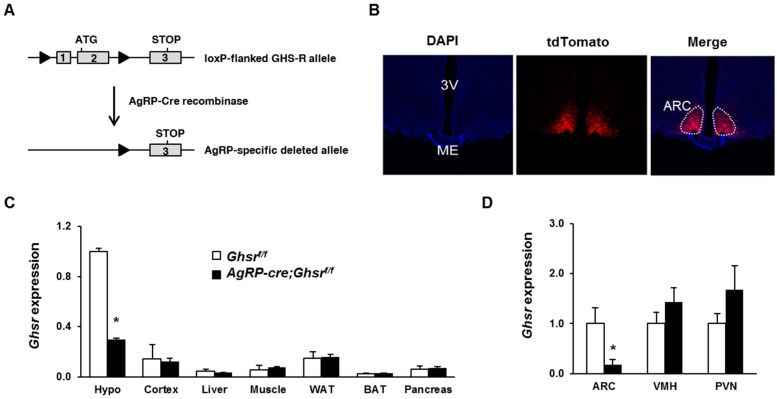
GHS-R in *AgRP-Cre*;*Ghsr^f/f^* mice is selectively deleted in arcuate nucleus of the hypothalamus. (**A**) Schematic diagram of the *loxP*-flanked *Ghsr* allele before and after Cre-derived recombination. Exons 1 and 2 were deleted during recombination. Triangle represents *loxP* sites; (**B**) Coronal section showing tdTomato-labeled neurons (red fluorescence) in the arcuate nucleus (ARC) in *Agrp-Cre;tdTomato* reporter mice; (**C**) *Ghsr* gene expression in hypothalamus, cortex, liver, skeletal muscle, epididymal white adipose tissue (WAT), brown adipose tissue (BAT) and pancreas; (**D**) *Ghsr* gene expression in ARC, ventromedial hypothalamus (VMH), and paraventricular nucleus (PVN) in the hypothalamus. * *p* < 0.05 compared between genotypes.

Mice with GHS-R deleted selectively in AgRP neurons (*AgRP-Cre*;*Ghsr^f/f^*) were generated by breeding a widely-used *AgRP-Cre* mouse [30,31] with our *Ghsr^f/f^* mouse [12]. The gene targeting strategy is shown in Figure 1A. To confirm the AgRP neuron-specific Cre activation, we crossed *AgRP-Cre* mice with the *Rosa26-tdTomato* mice; these mice express a red fluorescence protein following Cre-mediated recombination, thus enabling direct visualization of cells where recombination takes place (Figure 1B). In line with a previous report [31], AgRP-Cre-mediated recombination was only evident in hypothalamic arcuate nucleus (ARC) region, but was not evident in other brain regions. Indeed, real-time PCR results showed that *Ghsr* deletion was only detected in the hypothalamus, but not in other brain regions such as cortex, or peripheral tissues such as liver, muscle, white adipose tissue (WAT), brown adipose tissue (BAT) or pancreas (Figure 1C). Furthermore, we micro-dissected the arcuate nucleus (ARC), ventromedial hypothalamus (VMH) and paraventricular nucleus (PVN) from hypothalami of *AgRP-Cre;Ghsr^f/f^* and control *Ghsr^f/f^* mice, and then evaluated Ghsr expression. Consistent with the pattern of AgRP-Cre-mediated recombination, *Ghsr* expression was reduced by more than 80% in ARC, but not in other hypothalamic regions we examined (Figure 1D).

### 2.2. GHS-R Deletion in AgRP Neurons Abolishes Ghrelin-Induced GH Secretion and Food Intake

**Figure 2:**
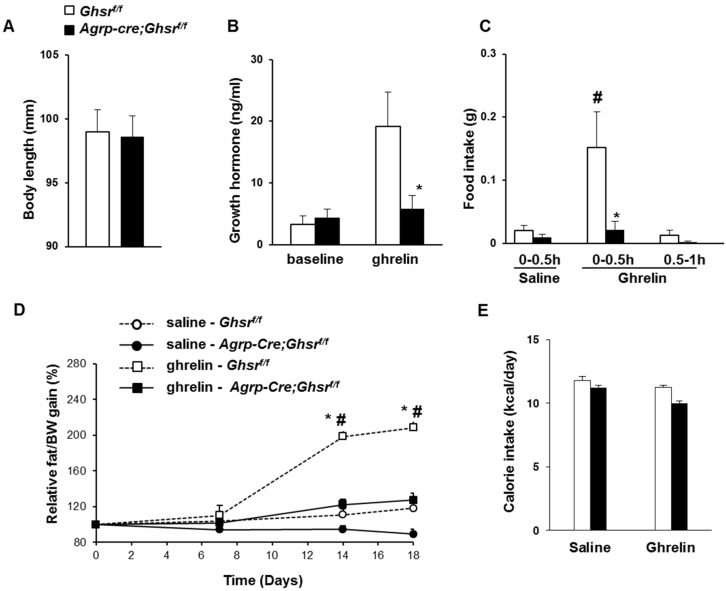
Ghrelin-induced GH secretion and acute food intake is abolished in *Agrp*-*Cre*;*Ghsr^f/f^* mice. (**A**) Body length of *AgRP-Cre*;*Ghsr^f/f^* and control *Ghsr^f/f^* mice; (**B**) Ghrelin-induced GH release: *Ghsr^f/f^* mice and *AgRP-Cre*;*Ghsr^f/f^* mice were anesthetized with pentobarbital (50 mg/kg); 15 min later, 20 µg ghrelin was injected. Blood was collected for GH detection at 0 and 5 min after ghrelin injection. (*n* = 5–9, * *p* < 0.05, *Ghsr^f/f^* mice vs. *AgRP-Cre*;*Ghsr^f/f^* mice); (**C**) Ghrelin-induced acute food intake. Ghrelin (0.5 mg/kg) was i.p. injected into mice in the early morning after 3 h fasting. # *p* < 0.05, saline vs. 30 min after ghrelin injection in *Ghsr^f/f^* mice; * *p* < 0.05, *Ghsr^f/f^* vs. *AgRP-Cre*;*Ghsr^f/f^*; (**D**) Chronic ghrelin-induced adiposity: 4-month old *Ghsr^f/f^* mice and *AgRP-Cre*;*Ghsr^f/f^* mice were i.p. injected with ghrelin for 18 days (days 1–7, 33 µg/daily was given; days 8–18, 33 µg twice a day was given). Relative fat percentage gain, compared to the beginning of the treatment, was increased in ghrelin-injected *Ghsr^f/f^* from day 14 compared with *AgRP-Cre*;*Ghsr^f/f^* group. * *p* < 0.05 compared between genotypes; # *p* < 0.05 compared between treatment groups; (**E**) Daily caloric intake was comparable between saline- and ghrelin-treatment groups, as well as between different genotypes.

Ghrelin is known to stimulate GH release and food intake via GHS-R. However, we found that deleting GHS-R in AgRP neurons had no effect on body length (Figure 2A), and insulin-like growth factor-1 (IGF-1) levels were comparable between *AgRP-Cre*;*Ghsr^f/f^* and control *Ghsr^f/f^* mice (data not shown). IGF-1 is mainly secreted by the liver as a result of GH stimulation, and is often used as an indicator of GH levels, while measuring the pulsatile GH levels in circulation is technically difficult in mice [32,33]. To assess whether GHS-R in AgRP neurons mediates the acute effects of ghrelin on GH secretion and food intake, we i.p. injected ghrelin into anesthetized *AgRP-Cre*;*Ghsr^f/f^* and control *Ghsr^f/f^* mice. Ghrelin-induced GH release was abolished in *AgRP-Cre*;*Ghsr^f/f^* mice (Figure 2B). While ghrelin-induced acute increase of food intake was readily detectable in control *Ghsr^f/f^* mice, it was absent in *AgRP-Cre*;*Ghsr^f/f^* mice (Figure 2C). These data indicate that the stimulatory effects of ghrelin on GH release and acute food intake are mediated by the GHS-R in AgRP neurons.

### 2.3. GHS-R Deletion in AgRP Neurons Attenuates Ghrelin-Induced Obesity

It has been reported that central ghrelin infusion promotes adiposity without inducing hyperphagia, indicating that ghrelin-induced adiposity is independent from its orexigenic effect [34]. To determine whether ghrelin’s effect on adiposity is mediated through GHS-R in AgRP neurons, we i.p. injected ghrelin into 4-month-old male *AgRP-Cre*;*Ghsr^f/f^* and control *Ghsr^f/f^* mice for 18 days. We started a 7-day regimen in these mice with a daily dose of 33 µg (10 nmol) ghrelin i.p., which has been shown to increase body weight [35]. We observed no significant increase in body fat between ghrelin-injected mice versus saline-injected mice in either *AgRP-Cre*;*Ghsr^f/f^* or *Ghsr^f/f^* mice. From day 8 to 18, we then increased to 2 times of 10 nmol ghrelin per day. We subsequently detected a higher gain in fat percentage in ghrelin-treated *Ghsr^f/f^* mice than in saline treated *Ghsr^f/f^* mice from day 14 to 18 (Figure 2D). Relative gain in fat percentage of ghrelin-treated *AgRP-Cre*;*Ghsr^f/f^* mice was significantly lower than that of ghrelin-treated *Ghsr^f/f^* mice from day 14 to 18. Calorie intake was not statistically different between *AgRP-Cre*;*Ghsr^f/f^* and *Ghsr^f/f^* mice treated with either saline or ghrelin (Figure 2E), indicating that the difference in adiposity is independent of hyperphagia. Hence, our data demonstrate that GHS-R in AgRP neurons is required for ghrelin-induced adiposity, and ghrelin-induced adiposity is independent of its orexigenic action.

### 2.4. GHS-R Deletion in AgRP Neurons Does Not Affect Energy Homeostasis under Regular Diet Feeding

**Figure 3:**
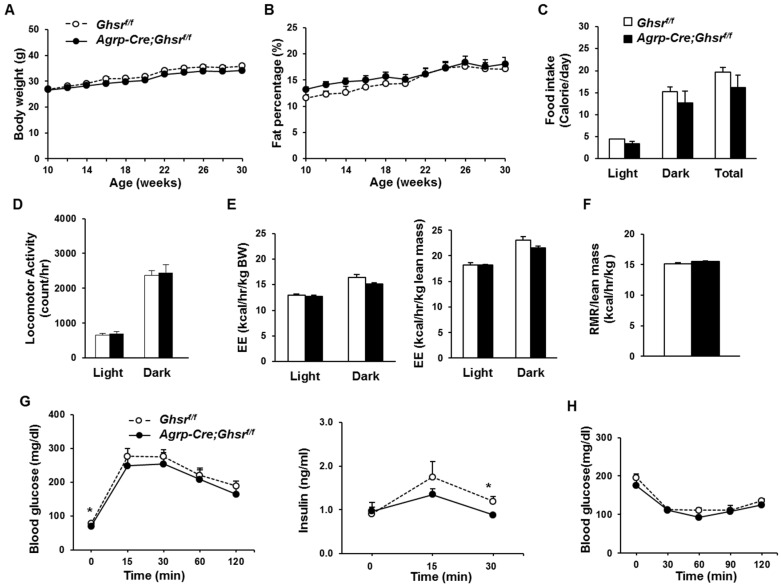
GHS-R deletion in AgRP-neurons does not affect energy homeostasis or insulin sensitivity in mice fed regular diet. (**A**,**B**) Body weight and fat percentage of *Ghsr^f/f^* and *AgRP-Cre;Ghsr^f/f^* mice, *n* = 10 and 15, respectively; (**C**–**F**) Indirect calorimetry analysis: (**C**) Daily food intake, (**D**) Locomotor activity, (**E**) Energy expenditure adjusted by body weight or lean mass, (**F**) Resting metabolic rate (RMR) normalized by lean mass; (**G**) Glucose and insulin levels during GTT after 18 h overnight fast. (**H**) ITT after 6 h morning fast. *n* = 6. * *p* < 0.05, *Ghsr^f/f^* vs. *AgRP-Cre;Ghsr^f/f^*.

To elucidate the effects of AgRP-specific GHS-R deletion on metabolism, we assessed body weight and body composition of regualr diet (RD)-fed *AgRP-Cre;Ghsr^f/^*^f^ mice and control *Ghsr^f/f^* mice. There were no significant differences in the body weight or fat content (genotype effect *F*_1,23_ = 1.262, *p* = 0.27 and genotype effect *F*_1,23_ = 0.542, *p* = 0.47, respectively) (Figure 3A,B). Furthermore, the metabolic assessment using Comprehensive Lab Animal Monitoring System (CLAMS) showed no significant difference between RD-fed *AgRP-Cre;Ghsr^f/f^* mice and control *Ghsr^f/f^* mice in food intake, locomotor activity, energy expenditure, or resting metabolic rate (Figure 3C–F). Together, these data suggest that GHS-R deletion in AgRP neurons does not have significant effects on energy homeostasis under normal feeding conditions.

We previously showed that ghrelin inhibits glucose-induced insulin secretion, and ghrelin ablation improves hyperglycemia of leptin-deficient mice [36]. We have also shown that global GHS-R ablation ameliorates age-associated obese and insulin-resistant phenotypes in old mice [11], demonstrating that GHS-R has important roles in energy- and glucose-homeostasis. To assess insulin sensitivity, we performed insulin tolerance tests (ITT) and glucose tolerance tests (GTT) on RD-fed *AgRP-Cre;Ghsr^f/f^* and control *Ghr^f/f^* mice. Fasting glucose levels of *AgRP-Cre;Ghsr^f/f^* mice were significantly lower at 0 time point (Figure 3G), but there was no significant difference in glucose excursions during GTT (genotype effect *F*_1,12_ = 0.6252, *p* = 0.44). Despite a significant decrease in insulin levels in RD-fed *AgRP-Cre;Ghsr^f/f^* mice at 30 min post bolus glucose injection in GTT, area under curve analysis showed no significant difference. No significant difference was detected in ITT (genotype effect *F*_1,12_ = 1.774, *p* = 0.21) (Figure 3H).

### 2.5. AgRP Neuron-Specific GHS-R Deletion Attenuates Diet-Induced Obesity

**Figure 4:**
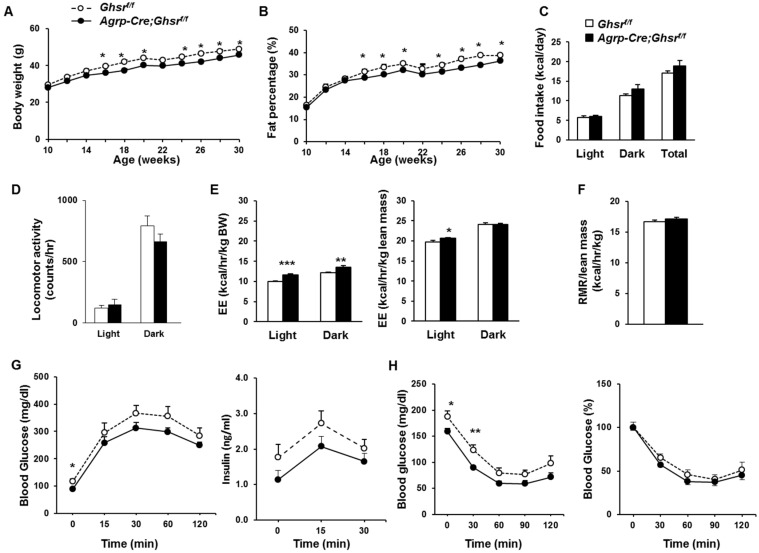
GHS-R deletion in AgRP-neurons mitigates DIO, showing increased energy expenditure. (**A**,**B**) Body weight and fat percentage of *Ghsr^f/f^* and *AgRP-Cre;Ghsr^f/f^* mice, *n* = 10; (**C**–**F**) Indirect calorimetry analysis: (**C**) Daily food intake, (**D**) Locomotor activity, (**E**) Energy expenditure adjusted by body weight or lean mass, (**F**) Resting metabolic rate (RMR) was measured during light cycle and normalized by lean mass; (**G**) Glucose and insulin levels during GTT after 18 h overnight fast; (**H**) ITT after 6 h morning fast. *n* = 6. * *p* < 0.05, ** *p* < 0.01, *** *p* < 0.001, *Ghsr^f/f^* vs. *AgRP-Cre*;*Ghsr^f/f^*.

We recently reported that deletion of GHS-R in all neurons effectively prevents DIO and significantly improves insulin sensitivity [12]. To determine whether GHS-R in AgRP neurons mediates the protective effect against DIO, we fed *AgRP-Cre*;*Ghsr^f/f^* and *Ghsr^f/f^* control mice with high-fat diet (HFD) starting at 10-weeks of age, and monitored changes in body weight, fat and lean mass (measured by Echo MRI) biweekly. Gains in body weight and fat content was significantly reduced in *AgRP-Cre*;*Ghsr^f/f^* mice compared to *Ghsr^f/f^* control mice, starting from 16 weeks of age (6 weeks after commencement of HFD feeding) (genotype effect *F*_1,22_ = 10.81, *p* = 0.003 and genotype effect *F*_1,22_ = 15.69, *p* = 0.0007, respectively) (Figure 4A,B). Metabolic assessment showed no difference in food intake or locomotor activity between *AgRP-Cre*;*Ghsr^f/f^* mice and control *Ghsr^f/f^* mice (Figure 4C,D), while energy expenditure was significantly increased in *AgRP-Cre*;*Ghsr^f/f^* mice compared to *Ghsr^f/f^* control mice (Figure 4E). Resting metabolic rate was not different between *AgRP-Cre*;*Ghsr^f/f^* and control mice (Figure 4F). Importantly, *AgRP-Cre*;*Ghsr^f/f^* mice exhibited increased energy expenditure, while physical activity and resting metabolic rate were unchanged. These data suggest that non-shivering thermogenesis is primarily responsible for the improved metabolic phenotype of *AgRP-Cre*;*Ghsr^f/f^* mice.

However, despite the lean phenotype observed in HFD-fed *AgRP-Cre;Ghsr^f/f^* mice, they did not show significant increase in insulin sensitivity compared to *Ghsr^f/f^* control mice. GTT showed that glucose levels of *AgRP-Cre;Ghsr^f/f^* were significantly lower at 0 time point (Figure 4G), but there was no significant difference in glucose excursions (genotype effect *F*_1,14_ = 2.332, *p* = 0.15). The glucose/insulin ratio was not significantly different between *AgRP-Cre;Ghsr^f/f^* and *Ghsr^f/f^* control mice, and area under curve analysis for insulin levels showed no significant difference.ITT showed significantly lower blood glucose levels in *AgRP-Cre;Ghsr^f/f^* after insulin administration at 0 and 30 min, but showed no difference when normalized to baseline glucose (genotype effect *F*_1,13_ = 1.987, *p* = 0.18) (Figure 4H).

### 2.6. AgRP Neuron-Specific GHS-R Deletion Enhances Thermogenesis

**Figure 5:**
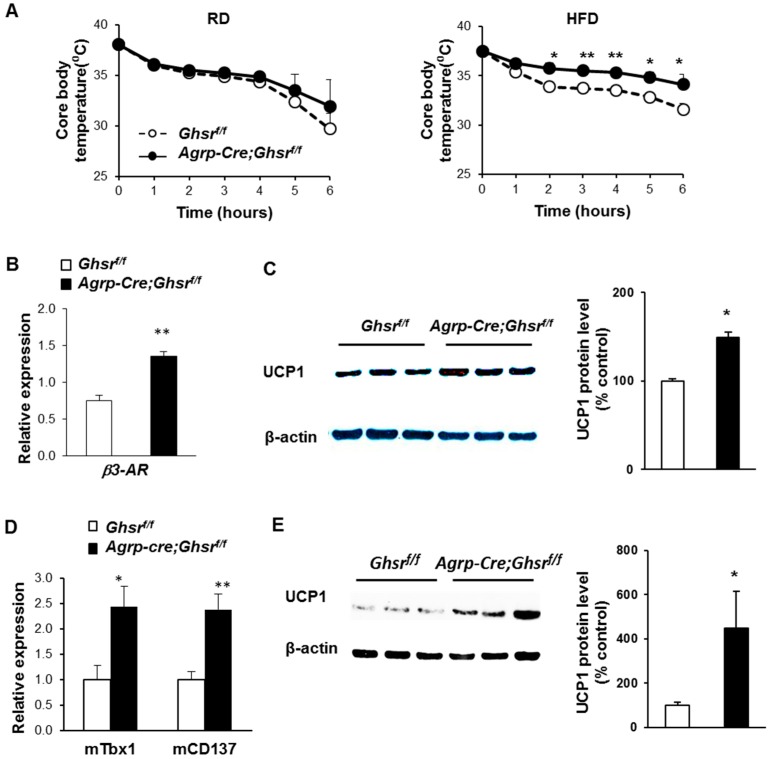
AgRP-neuron-specific GHS-R deletion increases thermogenesis, and activates thermogenesis in BAT and inguinal fat under HFD feeding. (**A**) Rectal temperature of RD- and HFD-fed mice during 4 °C cold exposure; (**B**) Expression of β3-adrenergic receptor gene in BAT; (**C**) Protein levels of thermogenic protein UCP1 in BAT; (**D**) Expression of beige adipocyte marker genes in inguinal fat; (**E**) Protein levels of thermogenic protein UCP1 in inguinal fat. Tissues were collected immediately after cold challenge for 6 h at 4 °C. *n* = 5. * *p* < 0.05, ** *p* < 0.001 *Ghsr^f/f^* vs. *AgRP-Cre;Ghsr^f/f^*.

The CLAMS analysis revealed that HFD-fed *AgRP-Cre;Ghsr^f/f^* mice have increased energy expenditure (Figure 4E). To determine whether increased energy expenditure in *AgRP-Cre;Ghsr^f/f^* mice is due to enhanced thermogenesis, we subjected both RD- and HFD-fed mice to 4 °C cold exposure for 6 h. While there was no difference in cold resistance between RD-fed *AgRP-Cre;Ghsr^f/f^* and control mice (genotype effect *F*_1,6_ = 0.6214, *p* = 0.4605), HFD-fed *AgRP-Cre;Ghsr^f/f^* mice exhibited higher cold-resistance than control mice, showing higher core body temperature (genotype effect *F*_1,7_ = 13.24, *p* = 0.008) (Figure 5A). Both brown adipocytes in brown adipose tissue (BAT) and beige adipocytes in subcutaneous fat possess thermogenic properties [37,38]. Indeed, we detected increased gene expression of β3-adrenergic receptor (*β3-AR*), and increased protein levels of the hallmark thermogenic regulatory protein, uncoupling protein-1 (UCP1), in BAT of HFD-fed *AgRP-Cre;Ghsr^f/f^* mice (Figure 5B,C). These data suggest that sympathetic nerve activity and thermogenic activity in BAT may be increased in HFD-fed *AgRP-Cre;Ghsr^f/f^* mice.

In addition to possible increased thermogenic function in BAT, the gene expression of beige adipocyte markers *Tbx1* and *CD137* were also increased in the inguinal fat of HFD-fed *AgRP-Cre;Ghsr^f/f^* mice, suggesting possible increased browning of inguinal fat (Figure 5D). Consistently, we also detected increased UCP1 protein levels in inguinal fat of HFD-fed *AgRP-Cre;Ghsr^f/f^* mice (Figure 5E). Together, these data suggest that the lean phenotype of HFD-fed *AgRP-Cre;Ghsr^f/f^* mice may be due to increased non-shivering thermogenesis in both BAT and WAT, since physical activity and resting metabolic rate were not altered in these mice.

### 2.7. Putative Regulators Invloved in GHS-R Associated Thermogenic Regulation

**Figure 6:**
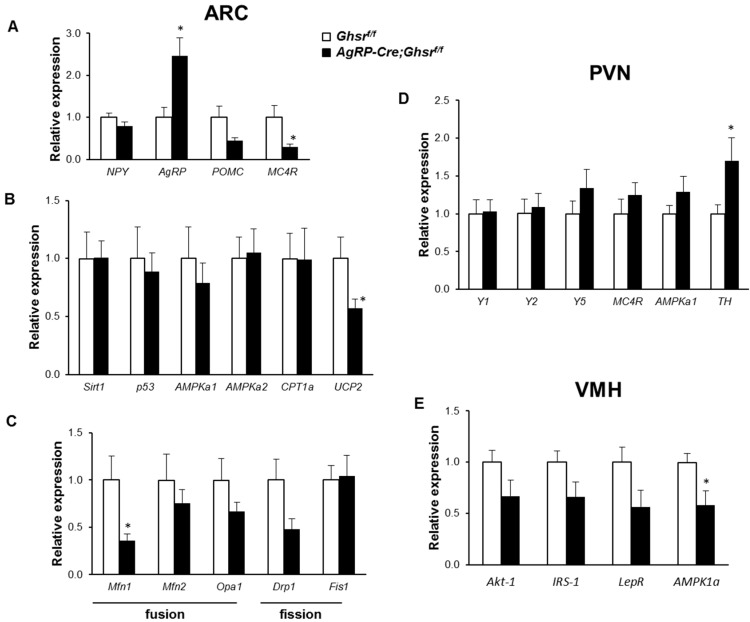
AgRP-specific GHS-R deletion alters gene expression in various hypothalamic regions in HFD-fed mice. Expression of various genes in ARC: (**A**) Expression of genes associated with orexigenic and anorexic signals; (**B**) Expression of key regulators known to be involved in mitochondrial function and β-oxidation; (**C**) Expression of genes involved in the mitochondrial dynamics. Expression of various genes in PVN (**D**) and VMH (**E**), supporting activated thermogenic signaling. Tissues were collected immediately after 6 h cold challenge at 4 °C. * *p* < 0.05, *Ghsr^f/f^* vs. *AgRP-Cre;Ghsr^f/f^*, *n* = 4–5.

To investigate the signaling network underlying the increased thermogenesis in HFD-fed *AgRP-Cre;Ghsr^f/f^* mice, we microdissected ARC from HFD-fed groups and analyzed gene expression of various signaling components. Given ghrelin’s role as an orexigenic hormone, we expected that deletion of GHS-R from AgRP neurons would lead to reduced expression of orexigenic signals. Intriguingly, while expression of orexigenic neuropeptide *Agrp* was significantly increased, expression of orexigenic *Npy* was not altered, in HFD-fed *AgRP-Cre;Ghsr^f/f^* mice compared to control *Ghsr^f/f^* mice (Figure 6A). In HFD-fed *AgRP-Cre;Ghsr^f/^*^f^ mice, the anorexic *Pomc* gene showed a trend of decrease in expression. Expression of melanocortin-4 receptor (*Mc4r*), the receptor for POMC-derived peptide α-MSH, showed a significant decrease (Figure 6A). In ARC, despite the increase of expression of orexigenic gene *Agrp* and the decrease of anorexic gene *Pomc*, the in vivo data showed no significant difference in long-term total food intake (Figure 4C). Thus, these data suggest other compensatory mechanisms exist in ARC for modulating energy sensing and calorie intake.

The sirtuin 1 (SIRT1)-p53 pathway has been shown to mediate the orexigenic action of ghrelin [39,40]. The activation by ghrelin of the SIRT1-p53 pathway leads to increased AMP-activated protein kinase (AMPK) activity, causing changes in hypothalamic mitochondrial respiration, production of reactive oxygen species, activation of carnitine palmitoyltransferase 1 (CPT1) and uncoupling protein 2 (UCP2) [25,41]. We analyzed the expression of these genes in microdissected ARC to test whether the sirtuin 1/p53 pathway may mediate the thermogenic effect of GHS-R in AgRP neurons. Deletion of GHS-R in AgRP neurons did not change expression of *Sirt1*, *p53*, *AMPKa1*, *AMPKa2*, or *Cpt1a*, whereas *Ucp2* was significantly decreased in HFD-fed *AgRP-Cre;Ghsr^f/f^* mice compared to control *Ghsr^f/f^* mice (Figure 6B). Recent findings suggest that the mitochondrial dynamics are important in the regulation of nutrient utilization and energy expenditure [42,43]. Here, we found that mitochondrial fusion gene *mfn1* was significantly decreased in HFD-fed *AgRP-Cre;Ghsr^f/f^* mice, while other genes involved in mitochondrial dynamics were not significantly altered (Figure 6C). Together these data suggest that deletion of GHS-R in AgRP neurons may alter mitochondrial activity, which may in turn affect AgRP neuronal activity. AgRP neurons are GABAergic [44]; decrease in neuronal activity of AgRP nuerons may lead to reduced inhibition of its downstream targets such as POMC neurons and neurons in PVN and VMH of the hypothalamus.

### 2.8. Putative Downstream Regulators That May Mediate GHS-R Suppression-Induced Thermogenesis

PVN is an important site for the regulation of energy expenditure [45,46]. Recent study suggested that NPY/AgRP neurons control sympathetic output and thermogenic function in BAT via a relay of tyrosine hydroxylase (TH) neurons in the PVN [47]. TH is a rate-limiting enzyme involved in the biosynthesis of catecholamines, including dopamine, norepinephrine and epinephrine. Hence, we studied the expression of genes in the NPY/AgRP-PVN pathway. Consistent with the observation that *Npy* was not significantly altered in HFD-fed *AgRP-Cre;Ghsr^f/f^* mice (Figure 6A), the expression of its receptors *Y1*, *Y2* and *Y5* was unchanged in PVN (Figure 6D). Despite the significant increase of *Agrp* in ARC (Figure 6A), expression of its downstream effector *Mc4r* was not changed in PVN of HFD-fed *AgRP-Cre;Ghsr^f/f^* mice (Figure 6D). Notably, *TH* expression was significantly increased in PVN, suggesting catecholamine output to downstream targets may be increased.

**Figure 7:**
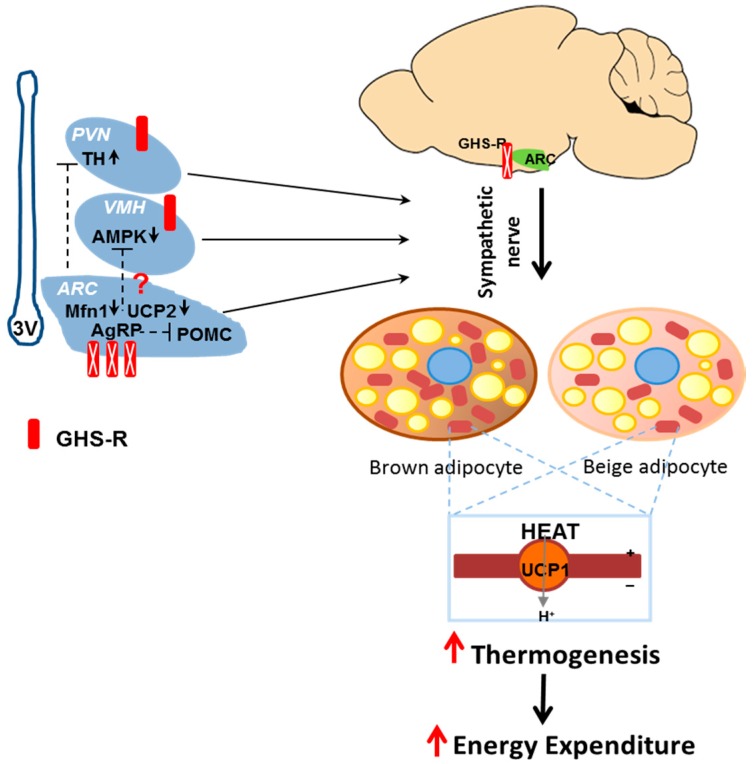
Schematic diagram of proposed neuronal circuits involved in AgRP-specific GHS-R mediated thermoregulation. Deletion of GHS-R in AgRP neurons may lead to decreased AgRP neuron activity, which decreases inhibitory tone to its downstream targets POMC neurons, PVN and VMH regions. GHS-R suppression in AgRP neurons may up-regulate the sympathetic outflow, which enhances thermogenesis in the brown and beige adipocytes, subsequently increasing energy expenditure (red upward arrows). Thus, GHS-R in AgRP neurons may regulate thermogenesis in BAT and WAT through ARC → PVN → sympathetic outflow, and/or ARC → VMH → sympathetic outflow pathways (Small black arrows in each brain regions indicate the gene expression changes in AgRP-specific GHS-R KO mice compared to WT mice: upward arrow = increase, downward arrows = decrease. Dotted line T bar shows the hypothetical inhibitory signals from AgRP neurons to POMC neurons in ARC or neurons in PVN and VMH. Red question mark shows the suggested axonal projections from AgRP neurons to VMH, which need to be confirmed).

VMH is another important site that controls energy expenditure, involving regulatory pathways such as AMPK-sympathetic nervous system and BAT axis-mediated thermogenic signaling [48,49,50]. Whole brain mapping of axonal projections from AgRP neurons suggest that AgRP neurons project to the neurons in VMH [51]. Interestingly, *AMPK1a* gene expression was decreased in VMH of *AgRP-Cre;Ghsr^f/f^* mice (Figure 6E). Leptin is an anorexic hormone, and leptin regulates thermogenesis via VMH [52]. However, expression of leptin receptor (*Lepr*) and its downstream mediator *STAT3* in VMH was not changed. Taken together, these gene expression data suggest that mitochondrial function may be altered in GHS-R deleted AgRP neurons. We hypothesize that GHS-R deficient AgRP neurons have reduced inhibitory tone to its downstream targets PVN neurons and VMH neurons, which may contribute to the concomitant increase of sympathetic nerve activity. These affected neuronal circuits may together lead to increased thermogenic activation in BAT and subcutaneous WAT, subsequently increasing energy expenditure. A schematic diagram depicting the hypothetical pathway is shown in Figure 7.

## 3. Discussion

In this study, we report that AgRP neuron-specific deletion of GHS-R increases energy expenditure. This increase is likely due to increased thermogenesis, as *AgRP-Cre*;*Ghsr^f/f^* mice show increased ability to maintain core body temperature during acute cold challenge, and increased expression of thermogenic marker UCP1 in BAT and WAT. AgRP neurons are a neuronal population located in the hypothalamic arcuate nucleus, and previous studies support a critical role of AgRP neurons in glucose-sensing and modulation of energy homeostasis [5,25,27,29,53,54]. Compelling evidence using optogenetic approaches showed that increased firing activity in AgRP neurons is sufficient to rapidly and robustly induce voracious feeding, even in satiated mice [54]. Ghrelin has been shown to directly activate AgRP neurons [5,25]. Re-expression of GHS-R in AgRP neurons, using tamoxifen-inducible *AgRP-CreER* in adult GHS-R null mice containing *floxed-STOP* codon preceding *Ghsr* gene, has been shown to partially restore orexigenic responses of ghrelin [27]. Acute administration of exogenous ghrelin leads to orexigenic stimulation, and chronic administration leads to fat deposition [1,7,55,56]. In this study, we showed that acute ghrelin-induced feeding was abolished in *AgRP-Cre;Ghsr^f/f^* mice, and chronic ghrelin-induced fat deposition was blunted in *AgRP-Cre;Ghsr^f/f^* mice. Interestingly, normal daily food intake and body length is not altered in *AgRP-Cre;Ghsr^f/f^* mice, suggesting compensatory mechanisms likely exist to maintain normal energy balance and growth under unchallenged condition. Furthermore, recent findings showed that ablation of ghrelin-producing cells in adult mice did not result in hypophagic phenotype and that ghrelin in normal physiological concentration ranges is not essential for regulation of food intake [57]. These results are in line with our finding that *AgRP-Cre;Ghsr^f/f^* mice have no reduction in total food intake.

Ghrelin and synthetic analogs are known to stimulate growth hormone (GH) release from the pituitary gland [58,59,60]. There is a clinical study showing that ghrelin promotes GH release mainly at the level of the arcuate nucleus [61]. It has been further demonstrated that ghrelin directly activates neurons of growth hormone-releasing hormone (GHRH) in the arcuate nucleus to stimulate GH secretion [62]. We have previously reported that acute administration of ghrelin to anaesthetized mice stimulates GH release [3]. Significant increase in serum GH levels was observed as early as five min post ghrelin injection, and this effect was reduced at 15 min post injection. Global deletion of GHS-R completely abolished ghrelin-induced GH release [3]. AgRP neurons have been reported to project directly to the pituitary to regulate GH-IGF-1 axis [63], but whether ghrelin directly activates AgRP neurons to stimulate GH secretion is not known. Here we used a similar approach as our previous publication [3], measuring serum GH levels before and 5 min post ghrelin injections in anaesthetized mice. We showed that acute ghrelin-induced GH release was abolished in *AgRP-Cre;Ghsr^f/f^* mice. While the anaesthetized condition helped to minimize confounding factors such as handling stress, other stress responses associated with blood sampling cannot be ruled out. Recent report shows that the newly improved GH hormone detection assay allows for reliable measurement of GH using as little as 2 µL of whole blood [64]. We will employ this new method in the future in examining pulsatile GH levels in mice. Nevertheless, our data provide direct in vivo evidence that GHS-R in AgRP neurons is required for ghrelin’s stimulatory effects on GH release.

Previously, we and others have reported that global GHS-R knockout mice show normal or slightly reduced body weights and fat mass when fed a regular chow diet [8,65]. Similarly, *AgRP-Cre*;*Ghsr^f/f^* showed no significant difference in body composition, food intake, energy expenditure and insulin sensitivity under RD feeding. However, when challenged with HFD, *AgRP-Cre*;*Ghsr^f/f^* mice showed increased energy expenditure, despite total energy intake being comparable. The increase in energy expenditure is likely due to increased non-shivering thermogenesis, since both physical activity and resting metabolic rate were similar. Consistent with the increased energy expenditure phenotype, *AgRP-Cre*;*Ghsr^f/f^* mice were more resistant to cold challenge, suggesting that GHS-R deletion in AgRP neurons may enhance thermogenesis.

Energy homeostasis is determined by the balance between energy intake and energy expenditure [66]. Enhancing thermogenesis to increase energy expenditure offers an attractive strategy to combat obesity. We previously reported that GHS-R plays an important role in BAT thermogenesis during aging, and GHS-R ablation improves age-associated thermogenic impairment [9]. Here we showed that under DIO condition, deletion of GHS-R in AgRP neurons led to increased β3-adrenergic receptor expression and increased UCP1 protein in BAT, and also led to increased expression of beige adipocyte markers as well as increased UCP1 protein levels in subcutaneous fat. Our data collectively suggest that GHS-R in AgRP neurons may play a central role in regulating adaptive thermogenesis in BAT and WAT, and this effect is more pronounced under the obese and cold challenged conditions. Further experiments measuring sympathetic activity and BAT tissue temperature would be very beneficial to confirm enhanced thermogenesis in *AgRP-Cre*;*Ghsr^f/f^* mice.

The ARC is one of the key hypothalamic nuclei that regulate energy homeostasis, and it contains both anorexigenic POMC neurons and orexigenic NPY/AgRP neurons [46]. Food restriction causes significant increases in expression of NPY and AgRP in the ARC and decreases adaptive thermogenesis [67]; this suggests that NPY and AgRP in ARC, in addition to their classic orexigenic property, also have important roles in thermoregulation. We found a paradoxical increase in *Agrp* expression and decrease in *Mc4r* expression in ARC of *AgRP-Cre*;*Ghsr^f/f^* mice; this suggests a decreased melanocortinergic tone. Our data also showed decreased *Ucp2* and *mfn1* expression in ARC of *AgRP-Cre*;*Ghsr^f/f^* mice, which imply altered mitochondrial function. Whether these mitochondrial changes lead to decreased neuronal activity in AgRP neurons, which subsequently decrease GABAergic signal output of AgRP neurons to downstream target sites need further confirmation.

Whole-brain mapping of axonal projections from AgRP neurons suggest that AgRP neurons project to neurons in PVN and VMH, which are important sites for the regulation of energy expenditure [51]. Our recent report of total neuronal deletion of GHS-R showed that PVN and VMH might be important sites involved in thermoregulation [12]. In this study, we further assessed the neuro-circuits involved in GHS-R mediated thermoregulation using *AgRP-Cre*;*Ghsr^f/f^* mice. NPY/AgRP neurons in ARC have been shown to inhibit thermogenesis via suppression of tyrosine hydroxylase (TH) neurons in the PVN [47]. AMPK-sympathetic nervous system-BAT axis has been shown to mediate thermogenic signaling in VMH [48,49,50]. Consistently, we found that *TH* expression in the PVN of HFD-fed *AgRP-Cre*;*Ghsr^f/f^* mice was increased, and *AMPK1a* expression was decreased in VMH of *AgRP-Cre*;*Ghsr^f/f^* mice; this suggests decreased inhibitory tone from GHS-R deficient AgRP neurons. We hypothesize that GHS-R in AgRP neurons regulates thermogenesis in BAT and WAT through ARC → PVN → sympathetic outflow and/or ARC → VMH → sympathetic outflow pathway (Figure 7). Future functional studies are required to confirm our current findings. Further investigations of GHS-R-mediated thermoregulation in AgRP neurons and its downstream neuronal pathways would be important for gaining a full understanding of how ghrelin signaling regulates sympathetic nerve activity and adaptive thermogenesis.

Recent studies show that DIO causes ghrelin resistance, and ghrelin sensitivity can be restored with weight loss ([68,69]; reviewed by Zigman et al. [70]). The authors propose that ghrelin resistance may serve as a protective mechanism to restrict a higher body weight set point established during DIO. Consistent with this idea, our data show that *AgRP-Cre*;*Ghsr^f/f^* mice are more resistant to DIO, suggesting GHS-R deficiency in AgRP neurons may mimic the ghrelin resistance state induced by HFD feeding, setting body weight gain at a lower threshold [70].

## 4. Materials and Methods

### 4.1. Animals

We previously described the generation of fully backcrossed GHS-R floxed mice on C57BL background [12]. Using a Cre-Lox system, we generated AgRP neuron-specific GHS-R knockout mice by breeding *Ghsr^f/f^* mice with widely used AgRP neuron-specific *AgRP-Cre* mice [30,31]. Mice were housed in the animal facility of Baylor College of Medicine, maintained at ~75 ± 1 °F with 12 h light/dark cycles (lights on 6 a.m. to 6 p.m.) with free access to water and food. For the current study, age-matched male *Ghsr^f/f^* (WT) and *AgRP-Cre*;*Ghsr^f/f^* mice were fed diets from Harlan Teklad (Madison, WI, USA): regular diet (RD) 2920X, with the caloric composition of 16% from fat, 60% from carbohydrates, 24% from protein; or high-fat diet (HFD) TD88137, with the caloric composition of 42% from fat, 42.7% from carbohydrates, 15.2% from protein. Separate cohorts of mice were used to study ghrelin’s pharmacological effects on growth hormone, acute food intake and adiposity, and in vivo long-term RD and HFD feeding. All experimental procedures used were approved by the Baylor College of Medicine Institutional Animal Care and Use Committee (IACUC, AN-2770, 8/7/2014–1/9/2017), and all methods were performed in accordance with the relevant guideline and regulations.

### 4.2. Body Composition and Indirect Calorimetry Studies

Whole body composition was monitored using Echo MRI-100 whole-body composition analyzer (Echo Medical Systems, Houston, TX, USA) to assess fat and lean mass. All metabolic parameters data were obtained using an Oxymax (Columbus Instruments, Columbus, OH, USA) open-circuit indirect calorimetry system, as we have previously described [11,71]. Mice were individually housed in feeding chambers and given free access to powdered diets for 4 days for initial acclimatization, then transferred to metabolic chambers for indirect calorimetry testing for 5 days. Data presented are averages of the day 2 to 4 of indirect calorimetry recordings. On the last day, the mice were fasted starting from 6 a.m. (light on) to 6 a.m. the following day (24 h fast). Resting metabolic rate (RMR) was calculated from the average of the 3 lowest points of energy expenditure values between 10 a.m. to 2 p.m. during the fasting period. Energy expenditure data was normalized to both lean mass and body weight. Locomotor activity was measured on *x*- and *z*-axes (horizontal and vertical activities, respectively) using infrared beams to count the number of beam breaks during the recording period.

### 4.3. Glucose Tolerance Tests (GTT) and Insulin Tolerance Tests (ITT)

For GTT, overnight-fasted mice were i.p. injected with 2.0 g/kg d-glucose (Sigma, St. Louis, MO, USA). At 0, 15 and 30 min time points, blood was collected from tail vein. A drop of blood was used to measure glucose, and 25 µL of blood was collected into EDTA-coated capillary tubes for insulin analysis. Plasma insulin during GTT was measured using Mouse Insulin ELISA kit (Cat: 10-1247-10, Mercodia, Sweden) according to manufacturer’s instruction. For ITT, mice were fasted for 6 h starting at 8:00 a.m., and 1 U/kg Humulin (Eli Lilly Company, Indianapolis, IN, USA) was i.p. injected. Blood glucose was measured at different time points (0, 15, 30, 60, 120 min) using OneTouch Ultra blood glucose meter (LifeScan, New Brunswick, NJ, USA).

### 4.4. Brain Processing for Immunofluorescence Imaging

Mice were anesthetized with isoflurane, then intracardially perfused with 50 mL of 10% formalin following saline washout. Subsequently the brains were removed and postfixed overnight in 10% formalin. Later the brains were cryoprotected by immersion in 30% sucrose in PBS at 4 °C. 25 µm coronal sections were cut using freezing microtome, mounted onto silane-coated glass slides, and coverslipped with Mounting Shield with DAPI (Vector Laboratories, Burlingame, CA, USA). Images were taken with Leica Microscope using MM AF software (Buffalo Grove, IL, USA).

### 4.5. Brain Regions for Expression Analysis

Whole brains of mice were collected and frozen on dry ice. Hypothalamic brain regions were dissected from 500 µm-thick coronal sections using 21 or 19 G Neuro Punches (Fine Science Tools, Inc., Foster City, CA, USA) under a magni-focuser (Edroy Products Company, Inc., Nyack, NY, USA) as described previously [12]. Punched tissues were stored at −80 °C until further analyses.

### 4.6. Quantitative Real-Time PCR

To assess the mRNA gene expression, total RNA was isolated using TRIzol^®^ Reagent (Invitrogen, Carlsbad, CA, USA) or Rneasy Mini kit (QIAGEN) for tiny tissues, according to the manufacturer’s instructions. In order to eliminate genomic DNA contamination, RNA samples were treated with RNase-free DNase (Ambion, Austin, TX, USA). Reverse transcription was performed with Superscript III First Strand Synthesis System (Invitrogen, Carlsbad, CA, USA). Quantitative real-time PCR reactions were performed in duplicates with Biorad CFX384 model (Bio-Rad Lab., Hercules, CA, USA). Relative gene expression levels were normalized by 18S. GHS-R-1a primers are: forward primer 5′-GGACCAGAACCACAAACAGACA-3′, reverse primer 5′-CAGCAGAGGATGAAAGCAAACA-3′ [13]. This primer set flanks the intron, which helps to distinguish functional ghrelin receptor GHS-R-1a from truncated GHS-R-1b. The rest of the primer information can be found in our recent publication [12].

### 4.7. Growth Hormone Assay

Growth hormone assay was carried out in 3-month old male mice as we have previously described [3]. Briefly, fed mice were i.p. injected with 50 mg/kg pentobarbital. Fifteen min later, 20 μg ghrelin was i.p. injected to induce GH release. Blood samples (80 μL) were collected from the tail vein at 0 and 5 min after ghrelin administration. The plasma concentration of GH was determined using GH Rat/Mouse Hormone RIA kit (Millipore Corporation, Billerica, MA, USA).

### 4.8. Ghrelin-Induced Spontaneous and Chronic Food Intake

The effect of ghrelin on spontaneous food intake was measured as we have previously reported [3]. Mice were singly housed for food intake measurements. Briefly, after 3 h fast (7:00 to 10:00 a.m.), mice were i.p. injected with saline and food intake was measured. Regular chow pellets were pre-weighed and placed inside each cage in a cup, and weighed again at indicated times. Any spillage was taken into account in calculating food intake. After 30 min, the same mice were i.p. injected with ghrelin at 0.5 mg/kg body weight. Food intake was then monitored every 30 min for 90 min. For chronic ghrelin treatment, 4-months old *AgRP-Cre*;*Ghsr^f/f^* and control *Ghsr^f/f^* mice were singly housed in metabolic cages equipped with balances (Columbus Instruments, Columbus, OH, USA). Powdered regular chow was used, and food intake was continuously monitored by the system, taking spillage into account. Mice were i.p. injected with ghrelin for 18 days (day 1 to 7, 33 µg (10 nmol)/daily; day 8 to 18, 33 µg twice daily). Daily body weight, fat mass and lean mass were assessed using Echo MRI body composition analyzer.

### 4.9. Cold Challenge Study

Core body temperatures were measured using a TH-8 Thermalert monitoring thermometer with a rectal probe (Physitemp Instruments Inc., Clifton, NJ, USA). Mice were individually caged for 6 h at 4 °C with free access to food and water. Body temperature was assessed hourly for 6 h; mice were then sacrificed immediately, and tissues were dissected, snap frozen and stored at −80 °C for further analysis.

### 4.10. Western Blot Analyses

About 0.2 g of brown adipose tissue (BAT) and inguinal white adipose tissue (ING) were sonicated in 1X RIPA Buffer containing complete Phosphatase Inhibitor Cocktail (PhosSTOP) and Protease Inhibitor Cocktail (Roche Diagnostics GmbH, Mannheim, Germany). Protein concentrations of samples were determined using BCA Protein Assay kit (Pierce, Rockford, IL, USA). 20 and 40 µg of proteins from BAT and ING were separated by 10% SDS-PAGE followed by electrophoretic transfer to a nitrocellulose membrane. Membranes were blocked in Tris-buffered saline with Tween 20 (TBS-T, 50 mM Tris-HCl (pH 7.5–8.0), 150 mM NaCl and 0.1% Tween 20) containing 5% non-fat milk for 1 h at room temperature, and incubated with anti-UCP1 (ABCAM, Cambridge, MA, USA, Ab10983, 1:10,000), or anti-β-actin (Cell Signaling, Danvers, MA, USA, 4967S, 1:1000) overnight at 4 °C. Blots were washed 3 × 15 min in TBS-T, followed by incubation in horseradish peroxidase-conjugated anti-rabbit secondary antibody (GE Healthcare Bio-Sciences, Pittsburgh, PA, USA, 1:10,000). This was followed by another 3 × 15 min washing in TBS-T, and visualization using the Pierce ECL Western Blotting Substrate. Densitometry analyses were performed using NIH ImageJ software (version 1.4, Bethesda, MD, USA).

### 4.11. Statistical Analysis

Data were presented as mean ± SEM. Graph-Pad Prism version 6.0 software (La Jolla, CA, USA) was used, and *p* < 0.05 was considered statistically significant. Two-way ANOVA with repeated measures were used to analyze body weight, fat percentage, GTT, ITT and cold stress data, and Sidak’s multiple comparisons test was used for post-hoc analysis. Student’s *t*-tests were used to compare genotype effects in all other experiments.

## 5. Conclusions

In summary, we found that GHS-R in AgRP neurons is essential for ghrelin-induced GH release, acute food intake and adiposity, but it is not required for long-term growth or total food intake. Important to note, AgRP neuron-specific deletion of GHS-R results in increased energy expenditure, likely due to increased thermogenic function in BAT and subcutaneous WAT, leading to decreased susceptibility to diet-induced obesity. Our results show that GHS-R in AgRP neurons has a key role in thermoregulation, which likely contributes to pathogenesis of DIO. The new model of *Agrp-Cre*;*Ghsr^f/f^* mice is a powerful tool that can be used to further investigate the neural circuits involved in adaptive thermogenesis. This study advances our understanding of the neural circuitries that mediate the metabolic effects of ghrelin signaling in hypothalamus, offers new insights for thermoregulation and energy metabolism at large, and also provides a novel anti-obesity strategy.

## Abbreviations

AgRP	Agouti-related peptide
ARC	Arcuate nucleus
BAT	Brown adipose tissue
DIO	Diet-induced obesity
GH	Growth hormone
GHS-R	Growth Hormone Secretagogue Receptor
HFD	High fat diet
NPY	Neuropeptide Y
PVN	Paraventricular nucleus of hypothalamus
SNS	Sympathetic nervous system
TH	Tyrosine hydroxylase
UCP1	Uncoupling protein-1
VMH	Ventromedial hypothalamus
WAT	White adipose tissue
